# Repeatability and Reproducibility of Quantification of Superficial Peri-papillary Capillaries by four Different Optical Coherence Tomography Angiography Devices

**DOI:** 10.1038/s41598-018-36279-2

**Published:** 2018-12-14

**Authors:** Jianqin Lei, Cheng Pei, Chan Wen, Nizar Saleh Abdelfattah

**Affiliations:** 1 Department of Ophthalmology, 1st affiliated hospital of Xi’an Jiaotong University, Xi’an, Shaanxi China; 2 Doheny Image Reading Center, Doheny Eye Institute, Los Angeles, CA USA; 3 Department of Ophthalmology, David Geffen School of Medicine at UCLA, Los Angeles, CA USA

## Abstract

This study was performed to test the repeatability and reproducibility of measurements of peri-papillary capillaries from four optical coherence tomography angiography (OCTA) devices. 109 healthy eyes were imaged with four OCTA devices (Spectralis, Optovue, Triton and Cirrus). A 3 × 3 mm scan pattern centered on the disc was repeated twice by each device. En face images of superficial capillary plexus were screened and processed for calculation. Vessel length density (VLD) was calculated on four equally divided parts of a ring between two concentric circles manually centered on the disc. General linear model (GLM) was used to test the impact of device and location on VLD. Intraclass correlation coefficient (ICC) of VLD between repeated scans was calculated. Of 218 acquisitions, 36%, 92%, 76% and 88% were eligible for analysis from Spectralis, Optovue, Triton and Cirrus, respectively. ICC was 0.94, 0.90, 0.84 and 0.87 for the four devices. GLM showed measurements significantly varied among devices (P < 0.001) and locations (P < 0.001). Pairwise comparison showed Triton = Spectralis >Optovue >Cirrus, and temporal = nasal >superior = inferior in measuring capillary VLD. This study revealed the repeatability of measuring peri-papillary capillaries was high for all four devices, while the reproducibility among the machines was unfavorable.

## Introduction

Optical coherence tomography angiography (OCTA) is a recently developed non-invasive technique that can display retinal vasculature with higher resolution and contrast compared to traditional dye-based angiography^1^. In only about three years, this new technique has been applied in a broad spectrum of macular diseases, as well as in optic neuropathies^2,3^. The enhancementand quantification of microvasculature around or on the optic disc broughtus further insights in diagnosing glaucoma and other optic neuropathies^3^. Many studies have reported on the measurement of peri-papillary capillaries in normal and glaucomatous eyes^4,5^, and the value of OCTA imaging in diagnosing glaucoma has been evaluated on different types of OCT devices^6–8^. The instruments from different manufacturers employ different algorithms that generate flow motion contrast images. However, the inter-instrument reproducibility in measurement of peri-papillary capillaries remains unknown.

Angiovue (RTVue XR Avanti, Optovue, Inc. Fremont, CA) was the first commercially available OCTA, usinga split spectrum amplitude decorrelation angiography (SSADA) algorithm withA-scan rate of 70 kHz. Angioplex (Cirrus HD-5000, Zeiss Meditec. Dublin, CA)was based on an optical microangiography (OMAG) algorithm with A-scan rate of 68 kHz. Triton (Topcon DRI OCT Triton, swept source OCT, Topcon, Japan.) employed an OCT angiography rate analyses (OCTARA) algorithm with A-scan rate of 100 kHz and Spectralis OCT2 module (Heidelberg Engineering, Germany)used an algorithm called full spectrum amplitude decorrelation with A-scan rate of 85 kHz. Munk *et al*.^9^ compared the above four instruments and reported a similar vessel density in macular superficial and deep capillary plexus among the four devices, while Shiihara *et al*.^10^ demonstrated the measurement of foveal avascular zone was significantly different among Triton, Angioplex and Angioscan (RS-3000 advanced OCT, Nidek Co. Ltd. Japan). By far, no data was available on the comparison of different OCTA devices in measurement of pericapillary vasculature. This is particularly important if different OCTA devices are involved in a multi-center clinical trial for whatever disease affecting peri-papillary capillaries.

## Results

### Demographics

**Figure 1:**
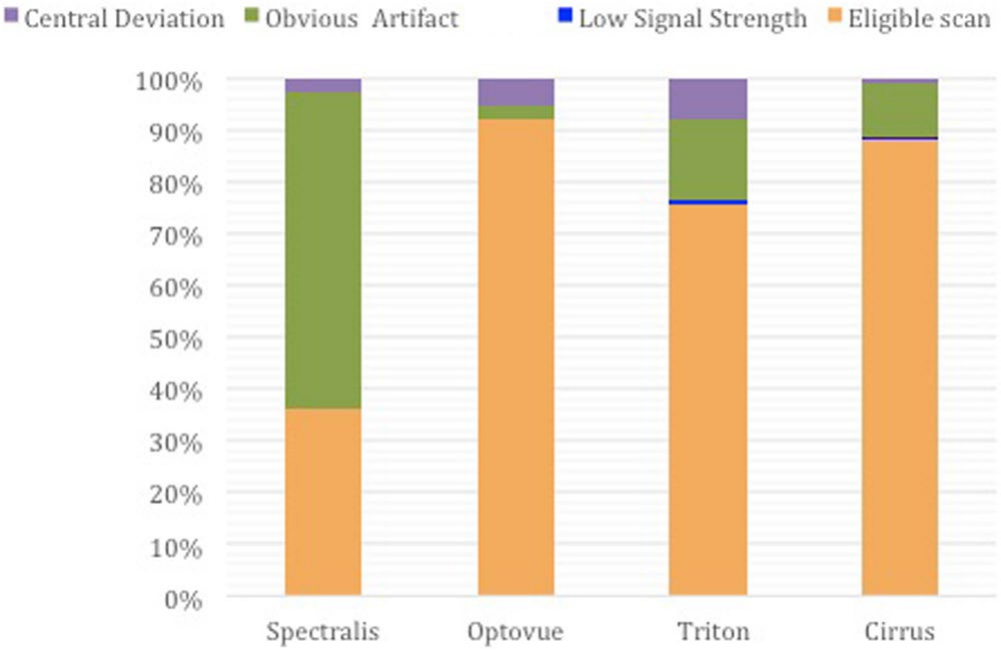
The proportion of excluded acquisitions by different reasons on the four OCTA devices. Only images from eligible scans were adequate for evaluation in this study.

One hundred and nine eyes from 109 participants (85 females, 109 Asians) receivedall 8 acquisitions on the four instruments. The mean age was 26 ± 6 (19–50) years and the mean axial length was 25.1 ± 1.3 (21.8–28.8) mm. Among the 218 scans, 36% (79/218), 92% (201/218), 76% (165/218) and 88% (192/218) were eligible for analysis from Spectralis, Optovue, Triton and Cirrus, respectively. Scans were excluded mainly from obvious artifacts. The reasons for excluded scans on each instrument areshown in Fig. 1.

### Reproducibility

**Figure 2:**
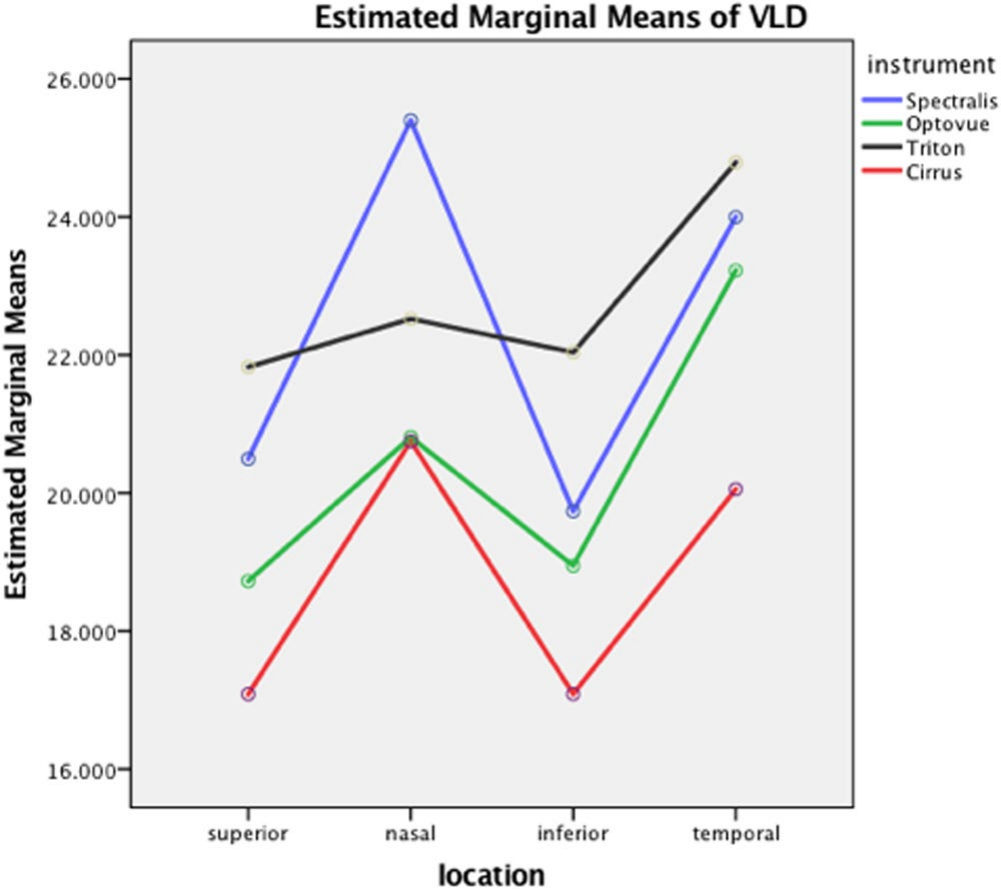
The estimated marginal means of vessel length density measured by four OCTA devices on four different locations.

For the comparative study, we had achieved at least one adequate image from each of the four machines on 39 eyes (31 females). The mean age was 27 ± 8 (21–50) years and the mean axial length was 24.8 ± 1.1 (21.8–26.6) mm, with 11 eyes included in group 2 (AL ≥ 25.5 mm). The mean VLD and the CV among individuals on the four locations for each instrument was listed in Table 1. The general linear model showed that instrument (F = 69, P < 0.001) and location (F = 35, P < 0.001) had significant impact on the measurement of VLD of peri-papillary capillaries, while AL did not affect the outcome measurements significantly. Pairwise comparison in this model demonstrated that Triton and Spectralis detected the most skeletonized vessels, followed by Optovue, and Cirrus detected the least (Table 2). The estimated means of VLD measured by each instrument on different locations is displayed in Fig. 2. The measurements approximated in superior and inferior peri-papillary area, which were significantly less than that in the temporal and nasal area, which were also similar (Table 2). The CVs and ICCs between each pair of instruments on the four locations are shown in Table 3.

**Table 1:** The mean values of capillary vessel length density and their coefficient of variation among individuals for each instrument by 4 different locations of a ring between two circles (1.5 and 2.25 mm) centered on the disc.

Location		Superior	Nasal	Inferior	Temporal
instrument					
Spectralis	mean ± SD (mm^−1^)	20.1 ± 3.4	25.2 ± 3.4	19.4 ± 3.1	23.8 ± 4.0
	CV (%)	17	14	16	17
Optovue	mean ± SD (mm^−1^)	18.8 ± 1.9	20.9 ± 2.3	18.7 ± 2.1	22.8 ± 2.3
	CV (%)	10	11	11	10
Triton	mean ± SD (mm^−1^)	21.9 ± 2.6	22.8 ± 2.9	21.9 ± 2.0	24.3 ± 2.7
	CV (%)	12	13	9	11
Cirrus	mean ± SD (mm^−1^)	16.9 ± 1.9	20.7 ± 2.4	16.8 ± 2.2	19.8 ± 4.4
	CV (%)	11	12	13	22

**Table 2:** Pairwise comparison of peripapillary vessel length density among different instruments and locations based on estimated marginal means using a general linear model.

Pairs	Mean difference of VLD (mm^−1^)	P value
Spectralis vs Optovue	1.98	<0.001
Spectralis vs Triton	−0.39	0.339
Spetralis vs Cirrus	3.66	<0.001
Optovue vs Triton	−2.37	<0.001
Optovue vs Cirrus	1.68	<0.001
Triton vs Cirrus	4.05	<0.001
Superior vs Nasal	−2.84	<0.001
Superior vs Inferior	0.08	0.835
Superior vs Temporal	−3.49	<0.001
Nasal vs Inferior	2.92	<0.001
Nasal vs Temporal	−0.65	0.250
Inferior vs Temporal	−3.57	<0.001

**Table 3:** Reproducibility of the measurement of vessel length density between each pair of the four OCTA devices on four different peripapillary locations.

Location		Superior	Nasal	Inferior	Temporal
Pairs of instruments					
Spectralis vs Optovue	CV (%) mean ± SD	10 ± 7	15 ± 8	10 ± 7	12 ± 9
	ICC (95% CI)	0.47 (0.00–0.72)	0.46 (−0.02–0.72)	0.36 (−0.23–0.66)	0.07 (−0.78–0.51)
Spectralis vs Triton	CV (%) mean ± SD	11 ± 9	11 ± 9	12 ± 10	9 ± 9
	ICC (95% CI)	0.40 (−0.15–0.69)	0.42 (−0.09–0.70)	0.10 (−0.72–0.53)	0.45 (−0.04–0.71)
Spectralis vs Cirrus	CV (%) mean ± SD	14 ± 8	16 ± 7	11 ± 7	15 ± 12
	ICC 95% CI	0.61 (0.26–0.80)	0.52 (0.08–0.75)	0.73 (0.48–0.86)	0.79 (0.61–0.89)
Optovue vs Triton	CV (%) mean ± SD	11 ± 5	7 ± 4	11 ± 7	6 ± 4
	ICC 95% CI	0.85 (0.71–0.92)	0.89 (0.79–0.94)	0.73 (0.48–0.86)	0.83 (0.67–0.91)
Optovue vs Cirrus	CV (%) mean ± SD	8 ± 6	5 ± 4	10 ± 6	15 ± 15
	ICC 95% CI	0.75 (0.52–0.87)	0.81 (0.64–0.90)	0.64 (0.31–0.81)	0.17 (−0.58–0.57)
Triton vs Cirrus	CV (%) mean ± SD	18 ± 9	9 ± 6	19 ± 9	17 ± 15
	ICC 95% CI	0.58 (0.20–0.78)	0.75 (0.52–0.87)	0.55 (0.14–0.76)	0.47 (−0.02–0.72)

### Correlation between axial length and VLD

Pearson’s correlation tests (Table 4) showed that the axial length was positively correlated with vessel length density on temporal peri-papillary area for both Optovue and Triton. There was a weak positive correlation on superior location for Spectralis and on inferior location for Cirrus.

**Table 4:** Correlation between axil length and the measurements on different locations for each device.

	Pearson correlationsig. (2-tailed)	Superior	Nasal	Inferior	Temporal
Spectralis	R	0.320	0.220	0.294	0.139
	P	0.047	0.177	0.070	0.398
Optovue	R	−0.006	−0.015	0.284	0.500
	P	0.973	0.929	0.079	0.001
Triton	R	−0.034	−0.115	0.268	0.538
	P	0.840	0.486	0.100	<0.001
Cirrus	R	0.241	0.067	0.337	0.148
	P	0.139	0.685	0.036	0.370

### Repeatability

In the repeatability test, 20 eyes had adequate images for analysis on both repeated scans from all the four instruments. The ICC was 0.94 (95% confidence interval (CI), 0.86–0.98), 0.90 (95% CI, 0.74–0.96), 0.84 (95% CI, 0.59–0.94) and 0.87 (95% CI, 0.67–0.95) for Spectralis, Optovue, Triton and Cirrus, respectively.

## Discussion

This study showed that all the four examined OCTA devices had high repeatability, suggesting their reliability in measuring peri-papillary capillaries, provided that adequate quality and centration of image was achieved. Generally speaking, the CVs between pairs of instruments were approaching or even higher than that among participants, indicating a low reproducibility among different types of machines.

In previous studies, the repeatability between scans has been reported to be high on individual commercialized instruments^11–15^. However, few studies compared their repeatability on the same cohort. It turned out in our study that the four instruments did not vary significantly in terms of repeatability (95% confidence interval of ICC overlapped). Although the ICC of Spectralis is the highest (0.94), variation of measurements among individuals was also the highest among the four machines, which might contribute to the high ICC. We also noticed that the proportion of eligible images was quite low for Spectralis. The main explanation maybe the long acquisiting time, which could lead to more motion artifacts and blinks. Besides, the participants would feel too stressed to cooperate well on Spectralis after two repeated scans, when the technician wished to get more scans if the previous one was unsatisfied. Another reason is that large patch of dark shadow was more frequent for Spectralis probably due to severe segmentation error or blink.

Munk *et al*.^9^ reported a similar macular superficial vessel density measured from those four instruments. However, although we examined exactly the same area on the four devices, there were still large disparities among them, and there was no overall good agreement among them. Thus, we suggest using the same instrument in future multi-center studies on peri-papillary capillaries. We believe the main reasons are the different calculating algorithms and segmentations of the peri-papillary superficial layer for each OCTA device. The definitions of the segmented layers for each machine varied somehow. But we didn’t manually adjust the segmentation, so that it provided a real-world data. When looking into each device closely, we found that the measurement from Spectralis disagreed with the remaining devices, so did the measurement between Triton and Cirrus. Agreement between Optovue and Triton was fairly good on each of the four locations (ICC ranging from 0.75 to 0.89), which could result from a similar definition of the segmentation. Agreement between Optovue and Cirrus on superior and nasal area was also acceptable, but not on temporal or inferior area. Noticeably, the peri-papillary retinal atrophy at the temporal and inferior side of the disc margin was not uncommon in this cohort because of the myopic shift. In some cases, the two segmentation lines overlapped in this atrophic zone for Cirrus and Spectralis resulting in a dark area. However, for Optovue and Triton, the lower segmentation boundary was a fixed offset below the ILM, which could result in the detection of choroidal capillaries and increased vessel density in this zone. This may also explain why there was a large variation among healthy participants on the temporal location for Cirrus (CV = 22%). These findings might be meaningful for future OCTA studies, especially on Asian population, due to the high prevalence of myopia.

Overall, Triton and Spectralis detected more skeletonized capillaries and Cirrus detected the least. We did not use the capillary perfusion density (PD) in this study because a blurred image or a decreased resolution might give a false perception of increased vessel density, which has been demonstrated in a study comparing the 3 × 3 mm and the 6 × 6 mm modes using Angioplex^12^. The PD within the central 3mm-ring measured by 6 × 6 mm mode was higher than that by 3 × 3 mm mode, while the VLD was lower in the same area by 6 × 6 mm mode. In our study, we used a multi-scale Hessian filter to decrease the noise and also eliminated the influence of large vessels. Thus, a higher amount of VLD could result from a better ability in detecting capillaries. More interestingly, the sequence of the amount of VLD seemed to be in accordance with the sequence of the A-scan rate for the four instruments.The swept source OCT with a longer wavelength and higher A-scan speed might have advantage in detecting peri-papillary capillaries. Previous studies have revealed that the OCTA with swept source and longer wavelength could define a larger area of choroidal neovascularization compared to a spectral domain OCTA^16,17^.

**Figure 3:**
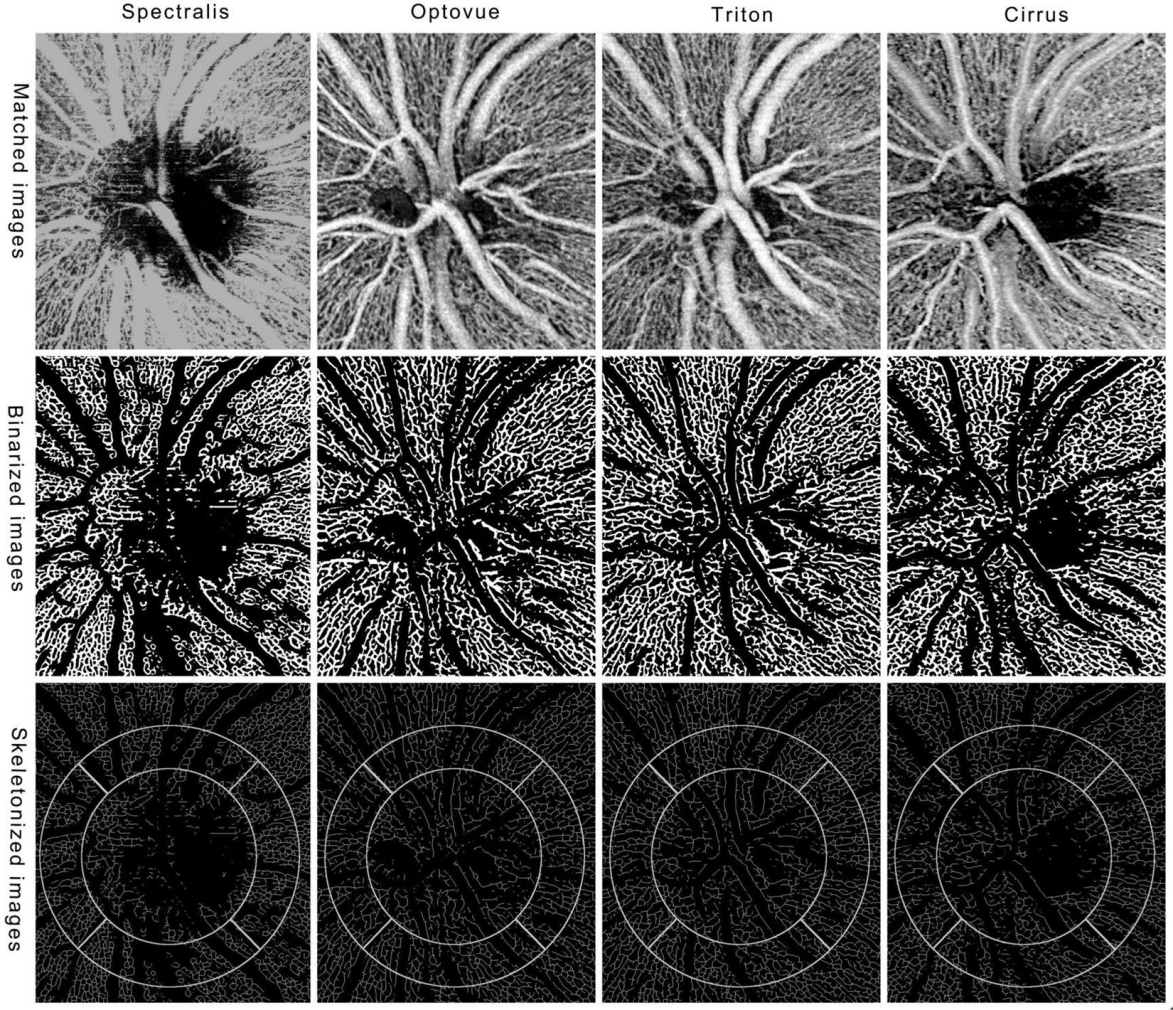
OCTA en face images of four devices. After registration, the four original images of four devices display exactly the same location (upper row). After a process of Hessian filter, auto-threshold and large vessel extraction, we got the binarized images (middle row). The images show single lines of retinal vessels after skeletonization and the selected locations for measurement are overlaid on the skeletonized images (bottom row). The diameters of inner and outer circles are 1.5 mm and 2.25 mm, respectively.

In this study, we also found out that the location significantly influenced the measurements, with higher VLD in the temporal and nasal peri-papillary areas, which could be explained by a lessdistribution of large vessels in these areas. From Fig. 3, we could see that the mean estimated VLD in the temporal area was higher than that in the remaining areas for Triton and Optovue. As we have mentioned above, choroidal capillaries could be segmented into the studied layer for eyes with peri-papillary atrophy.

Previous studies have demonstrated a negative correlation between peri-papillary vessel density and elongation of axial length^18–20^. In this study, we also tested the influence of axial length on all four different types of OCTA instruments. It turned out that the measurements were not significantly affected by variation of axial length. Compared to previous studies, we only included healthy participants, and only a small proportion of them have high myopia. Besides, eyes with obvious myopic maculopathy or with vitreous haze were excluded. When a specific peri-papillary location was evaluated separately, we found that the vessel length density at temporal side had a positive correlation with the axial length for Optovue and Triton, which might be explained by the enhancement of choroidal capillaries in this area with retinal atrophy commonly seen in myopic eyes. There was also a weak positive correlation on superior side for Spectralis and inferior side for Cirrus. We did not correct the magnification from elongation of the eyeball, which could result in higher calculation readings of the vessels.

There were several limitations of our study. One major concern is the measurement of vessels was based on the intensity of pixels. Though denoising was applied during image processing, it is still possible that the non-vascular signal was calculated as vessel density. Thus, a higher vessel length density does not necessarily mean a better resolution. Another limitation is the small sample size for the multiple comparison, although the sample was larger than previous comparative studies on different machines. Finally, the majority of the participants were young females, thereby we could not evaluate the influence of age or gender on the measurements of each device.

Despite those limitations, our study is not without strengths. We’ve taken strict measures to exclude images with inadequate quality in order to minimize the influence of artifacts that might lead to incorrect calculation. And that is why only 39 out of 109 eyes were finally included in the study. In conclusion, our study revealed that the repeatability was high for all the four instruments, reassuring their reliability in evaluation of peri-papillary capillaries. The reproducibility among the four instruments was unfavorable, while the agreement between Optovue and Triton was fairly good, usually with a higher measurement of VLD for the latter. Longer axial length ( ≥ 25.5 mm) did not influence the measurement. However, the increased VLD in the temporal peri-papillary area with longer axial length for Optovue and Triton could result from the atrophy in this area and choroidal capillaries could be segmented into this layer. Those findings may provide valuable information for future clinical trials and practices.

## Methods

### Study Design and Image Acquisition

In this cross-sectional study, healthy volunteers over 18 years of age with no history of systemic or eye diseases were recruited. Best corrected visual acuity of all eyes was at least 20/20. Eyes with posterior vitreous detachment and a Weiss’s ring anterior to the disc were excluded. High myopia was not listed in the exclusion criteria, however, the severity of myopic maculopathy should be no more than category 1 (tessellated fundus only)^21^. Any myopic retinal lesion that beyond category 1 or there is “plus” lesion^21^ was excluded. All studied eyes were divided into two groups according to their axial lengths (AL) (group 1, AL < 25.5 mm; group 2, AL ≥ 25.5 mm).

Of all enrolled participants, one eye was randomly chosen and was imaged using four OCTA devices in a random sequence. The four devices were Spectralis OCT2 module (version 6.8b), Angiovue (version 2016.2.0.35), Triton (OCTARA, version 1.0) and Angioplex (version 9.0). A 3 × 3 mm scan pattern centered on the disc was repeated twice on each eye. Four trained technicians designated by the Chinese agents of the four manufacturers performed the examinations on their own OCTA devices and acquisitions met a minimum demand of signal strength (25 for Spectralis, 50 for Angiovue, 50 for Triton and 7 for Zeiss). Axial length was examined using IOL Master (version 3.02, Carl Zeiss Meditec, Jena, Germany) for each study eye. Written informed consent was obtained from all individuals prior to image acquisitions. The study was approved by the Institutional Review Board of Xi’an Jiaotong University and conducted in accordance with the ethical standards stated in the Declaration of Helsinki.

### Image Processing

En face images of peri-papillary vasculature were generated automatically by each instrument software. Retinal slab of superficial capillary plexus (defined by the instrument as region spanning from the internal limiting membrane (ILM) to the inner plexiform layer on Spectralis and Cirrus) was used in the study. The slab of optic nerve head was used on Optovue (spanning from ILM to a boundary 150 μm below the ILM) and Triton (spanning from ILM to a boundary 130 μm below the ILM). No segmentation was manually corrected.

Image with inadequate quality was excluded if any of the following criteria was met. (1) there was obvious artifact identified as saccades (horizontal misalignment of vessel segments) ≥3, or at least one patch of dark shadow that obscures retinal vessels outside the disc area. (2) Disc central deviation prevented from achieving the target area after matching the four images from the different machines. (3) The signal strength was below the minimum demand (described above). A resident in ophthalmology (C.W.) went through all the images and screened for adequate ones according to the image exclusion criteria. Questionable images (the first grader had less than 90% of the confidence in judging the image quality) were reviewed by a retinal specialist (J.L.) and a final decision was made through open adjudication.

The scan with a better image quality from the two repeated acquisitions was included in the comparative study so that each study eye had four en face images of superficial retinal vasculature centered on the disc from the four different instruments. The four images were exported and transformed to the same size (1024 × 1024 pixels corresponding to 3 × 3 mm).

Image J software (U. S. National Institutes of Health, Bethesda, Maryland)was used for all image processing. Registration was conducted by manually matching four distinct anatomical features (for example, bifurcation of a certain vessel) so that the four images displayed exactly the same area (Fig. 3, upper row). The average intensity was also adjusted to the same level for the four images. Then, the handled image underwent Hessian filter (smoothing = 3) followed by an auto- threshold (Otsu) to get the initial binarized image. Large vessels were also extracted using Hessian filter (smoothing = 15) and auto-threshold (Otsu), followed by removing outliers (radius = 15). The large vessels were then subtracted from the initial binarized image to get the binarized image of peri-papillary capillaries (Fig. 3, middle row).

Skeletonization was further applied to get a single line of capillaries. Vessel length density (VLD) (defined as vessel length per unit area based on the skeletonized image) was calculated on four equally divided locations (superior, nasal, inferior and temporal) of a ring between two concentric circles (1.5 and 2.25 mm in diameter) manually centered on the disc (Fig. 3, bottom row).

We also tested the repeatability between the two consecutive scans on each of the four devices. No image registration was needed for this study. The VLD was calculated within the ring between the 3-mm and 1-mm circles centered on the image.

### Statistical Analysis

To test the general effect of different instruments on the outcome measurements, the general linear model (repeated measures) was used, with instrument and location being as within-subject effect and axial length being as between-subject effect. We also evaluated the association between axial length and the metrics on each location of the four instruments using Spearman correlation test. To test the reproducibility among different instruments, intraclass correlation coefficient (ICC)and coefficient of variation (CV) was calculated between each pair of instruments on each location. The CVs among healthy subjects were also calculated. For the repeatability test, ICC between the repeated scans on each device was calculated. SPSS 23.0 (IBM SPSS Statistics for Windows, Version 23.0. Armonk, NY) was used for all statistical analyses.

## Data Availability

The datasets generated during and/or analyzed during the current study are available from the corresponding author on reasonable request.

## References

[1] Spaide RF, Klancnik JM, Cooney MJ (2015). Retinal vascular layers imaged by fluorescein angiography and optical coherence tomography angiography. JAMA Ophthalmol..

[2] De Carlo TE, Romano A, Waheed NK, Duker JS (2015). A review of optical coherence tomography angiography (OCTA). Int. J. Retin. Vitr..

[3] Akil H, Falavarjani K, Sadda S, Sadun A (2017). Optical coherence tomography angiography of the optic disc; an overview. J. Ophthalmic Vis. Res..

[4] Jia Y (2014). Optical coherence tomography angiography of optic disc perfusion in glaucoma. Ophthalmology..

[5] Suh MH (2016). Optical coherence tomography angiography vessel density in glaucomatous eyes with focal lamina cribrosa defects. Ophthalmology..

[6] Shin JW, Sung KR, Lee JY, Kwon J, Seong M (2017). Optical coherence tomography angiography vessel density mapping at various retinal layers in healthy and normal tension glaucoma eyes. Graefes Arch. Clin. Exp. Ophthalmol..

[7] Chihara E, Dimitrova G, Amano H, Chihara T (2017). Discriminatory Power of Superficial Vessel Density and Prelaminar Vascular Flow Index in Eyes With Glaucoma and Ocular Hypertension and Normal Eyes. Invest. Ophthalmol. Vis. Sci..

[8] Akil H, Huang AS, Francis BA, Sadda SR, Chopra V (2017). Retinal vessel density from optical coherence tomography angiography to differentiate early glaucoma, pre-perimetric glaucoma and normal eyes. Plos One..

[9] Munk MR (2017). OCT-angiography: A qualitative and quantitative comparison of 4 OCT-A devices. Plos One..

[10] Shiihara H (2017). Reproducibility and differences in area of foveal avascular zone measured by three different optical coherence tomographic angiography instruments. Sci. Rep..

[11] Venugopal JP (2017). Repeatability of vessel density measurements of optical coherence tomography angiography in normal and glaucoma eyes. Br. J. Ophthalmol..

[12] Lei J (2017). Repeatability and Reproducibility of Superficial Macular Retinal Vessel Density Measurements Using Optical Coherence Tomography Angiography En Face Images. JAMA Ophthalmol..

[13] Coscas F (2016). Normative Data for Vascular Density in Superficial and Deep Capillary Plexuses of Healthy Adults Assessed by Optical Coherence Tomography Angiography. Invest. Ophthalmol. Vis. Sci..

[14] Al-Sheikh M, Tepelus TC, Nazikyan T, Sadda SR (2017). Repeatability of automated vessel density measurements using optical coherence tomography angiography. Br. J. Ophthalmol..

[15] Al-Sheikh M, Ghasemi Falavarjani K, Akil H, Sadda SR (2017). Impact of image quality on OCT angiography based quantitative measurements. Int. J. Retin. Vitr..

[16] Novais EA (2016). Choroidal Neovascularization Analyzed on Ultrahigh-Speed Swept-Source Optical Coherence Tomography Angiography Compared to Spectral-Domain Optical Coherence Tomography Angiography. Am. J. Ophthalmol..

[17] Zhang Q (2017). Automated Quantitation of Choroidal Neovascularization: A Comparison Study Between Spectral-Domain and Swept- Source OCT Angiograms. Invest. Ophthalmol. Vis. Sci..

[18] Sung MS, Lee TH, Heo H, Park SW (2017). Clinical features of superficial and deep peripapillary microvascular density in healthy myopic eyes. PLoS One..

[19] Wang X (2016). Is the peripapillary retinal perfusion related to myopia in healthy eyes? A prospective comparative study. BMJ Open..

[20] Mo J, Duan A, Chan S, Wang X, Wei W (2017). Vascular flow density in pathological myopia: an optical coherence tomography angiography study. BMJ Open..

[21] Ohno-Matsui K (2015). International photographic classification and grading system for myopic maculopathy. Am. J.Ophthalmol..

